# Traffic and Driving Simulator Based on Architecture of Interactive Motion

**DOI:** 10.1155/2015/340576

**Published:** 2015-09-30

**Authors:** Alexander Paz, Naveen Veeramisti, Romesh Khaddar, Hanns de la Fuente-Mella, Luiza Modorcea

**Affiliations:** ^1^University of Nevada, Las Vegas, Civil and Environmental Engineering and Construction, Las Vegas, NV 89154, USA; ^2^University of Nevada, Las Vegas, Electrical and Computer Engineering, Las Vegas, NV 89154, USA; ^3^Pontificia Universidad Católica de Valparaíso, Facultad de Ciencias Económicas y Administrativas, Escuela de Comercio, Avenida Brasil 2830, 2340031 Valparaíso, Chile; ^4^Faculty of Law, Transilvania University of Brasov, 500036 Brasov, Romania

## Abstract

This study proposes an architecture for an interactive motion-based traffic simulation environment. In order to enhance modeling realism involving actual human beings, the proposed architecture integrates multiple types of simulation, including: (i) motion-based driving simulation, (ii) pedestrian simulation, (iii) motorcycling and bicycling simulation, and (iv) traffic flow simulation. The architecture has been designed to enable the simulation of the entire network; as a result, the actual driver, pedestrian, and bike rider can navigate anywhere in the system. In addition, the background traffic interacts with the actual human beings. This is accomplished by using a hybrid mesomicroscopic traffic flow simulation modeling approach. The mesoscopic traffic flow simulation model loads the results of a user equilibrium traffic assignment solution and propagates the corresponding traffic through the entire system. The microscopic traffic flow simulation model provides background traffic around the vicinities where actual human beings are navigating the system. The two traffic flow simulation models interact continuously to update system conditions based on the interactions between actual humans and the fully simulated entities. Implementation efforts are currently in progress and some preliminary tests of individual components have been conducted. The implementation of the proposed architecture faces significant challenges ranging from multiplatform and multilanguage integration to multievent communication and coordination.

## 1. Introduction

A vast number of studies have illustrated the potential of driving simulators to analyze actual driver behavior for multiple purposes, such as traffic safety and information provision [1–5]. The history of driving simulators can be traced back to the 1920s, with research for various purposes [6]. In the 1980s, Daimler-Benz [7] developed a high-fidelity driving simulator, encouraging others to develop even better simulators. Several researchers and commercial companies have developed driving simulators ranging from fixed-based simulators to the most advanced motion-based simulators known today. Some of the newest driving simulators include the National Advanced Driving Simulator (NADS), funded by NHTSA and maintained by the University of Iowa [8]; the Driving Environment Simulator (DES), developed by the University of Minnesota [9] in collaboration with AutoSim and Realtime Technologies; the VTI Simulator IV by the Swedish National Road and Transport Research Institute [10]; the University of Leeds driving simulator [11]; DriveSafety driving simulator by the University of Michigan Transportation Research Institute (UMTRI) [12]; the STISIM Drive driving simulator by System Technology Inc.; [13] and the UC-win/Road driving simulator by FORUM8 [14].

The National Advanced Driving Simulator (NADS) Laboratory [8] at the University of Iowa has very advanced driving simulators including NADS-1, NADS-2, and the MiniSim simulator. NADS-1 is an advanced motion-based ground vehicle simulator. NADS-2 is similar to NADS-1 but fixed-based. The MiniSim is a PC-based, high-performance driving simulator that uses the same technology as NADS-1. However, MiniSim can be used at a lower cost than NADS-1 and NADS-2 because it is portable and easy to set up and operate.

The Human Factors Interdisciplinary Research in Simulation and Transportation Program (HumanFIRST) at the University of Minnesota has the Driving Environment Simulator (DES) [9]. DES is an immersive driving simulator that provides high fidelity simulation to generate a realistic presence within the simulated environment. DES measures psychophysiological responses, including brain activity—for example, Evoked Response Potential (ERP). DES also includes highly accurate eye-tracker software. HumanFIRST has a portable, low-cost driving simulator that uses the same technology as DES.

UC-win/Road [14] is a Virtual Reality (VR) environment where the driver can navigate in a three-dimensional (3D) space. The environment, including a traffic simulation and visualization tool, uses ground texture maps and can include 3D building images. The environment also includes traffic generation models to generate traffic on various lanes and roads.

This study proposes an architecture for an interactive motion-based traffic simulation environment. Although the existing driving simulation models provide tremendous capabilities to study driving behavior in a safe and controlled environment, there are multiple aspects of the real-world that can be addressed to significantly enhance modeling realism. The proposed architecture integrates multiple types of simulation, including (i) a motion-based driving simulation; (ii) a pedestrian simulation; (iii) a motorcycling and bicycling simulation; and (iv) a traffic flow simulation. This integration enables the simultaneous and interactive interaction between actual and simulated drivers, pedestrians, and bike riders. In addition, the architecture provides capabilities to simulate the entire network at a reasonable price; in this way, the drivers, pedestrians, and bike riders can navigate anywhere in the system.

To increase modeling realism, the proposed architecture enables actual humans to experience background traffic and the background traffic to be affected by the decisions and behavior of the actual humans navigating the system. To achieve this interaction, the background traffic is modeled using a hybrid mesomicroscopic modeling approach for traffic flow simulation. The mesoscopic traffic flow simulation module of the hybrid model loads the results of a user equilibrium traffic assignment solution and propagates the corresponding traffic throughout the entire system. The microscopic traffic flow simulation model provides background traffic around the vicinities where actual human beings are navigating the system. The two traffic flow simulation models interact continuously to update system conditions, based on the interactions between actual humans and the fully simulated entities. The interaction between actual and background traffic has tremendous implications. For example, in the real-world, an accident, as consequence of a human error, can affect a large portion of the traffic system. These types of scenarios of significant interest for a number of applications cannot be modeled realistically without using the proposed architecture that integrates the driving simulator and the microscopic traffic flow simulator. Such scenario requires capabilities to propagate traffic and represent congestion related phenomena including queue formation and dissipation as well as spillback and spillover. Existing driving simulation models do not provide such capabilities because they do not model background traffic using realistic traffic flow models.

Depending upon the nature of the experiment, a scenario can be designed using a single simulator or any instances of simulators. Some examples for which the proposed system can be used include the following:

1. The evaluation of driver and pedestrian behavior for traffic safety projects including distracted driving and/or walking and driving and/or walking under the influence of alcohol or drugs [1–5].
2. The evaluation of multiple mechanisms or technologies designed to assist the driving task and to improve traffic safety. Some of these mechanisms and technologies include crosswalks, flashing lights, infrared sensors to detect pedestrians, distance warning systems, dynamic cruise control, automatic braking systems, and dynamic navigation.
3. The simultaneous study of the interactions among drivers, pedestrians, and bikers as well as the interactions of them with the infrastructure and the control and information mechanisms and the different technologies used influencing behavior and/or assisting the navigation tasks. The existing literature [15–17] relies on models that do not use actual humans during the experimental part of the analysis.
4. The training of first responders and emergency personnel. Multiple potential emergency scenarios can be designed and used to provide adequate training in safe and controlled environment. Burke et al. [18] documents the importance of training such personnel. Accidents involving emergency vehicles were analyzed using several sources of information including video data as well as interviews. The results highlighted that drivers' errors were responsible for most of the accidents.

In this study, implementation efforts are currently in progress, and some preliminary tests of individual components have been conducted. The implementation of the proposed architecture faces significant challenges, ranging from multiplatform and multilanguage integration to multievent communication and coordination. To address some of those challenges and achieve the greatest benefits at the lowest cost, state-of-the-art technologies currently are being used to implement the proposed architecture. Some of these technologies include (i) Open Street Maps (OSM) [19]; (ii) Blender [20]; (iii) DynusT© [21]; CORBA; and free SDKs, such as MS Kinect [22] and Ardunio [23]. The proposed architecture is called Networked Motion-Based Interactive PEdestrian and Driving Simulator (n-MIPEDS). Although particular suggestions to implement the proposed architecture are provided in this paper, the conceptual architecture is general and can be implemented using multiple technologies. Appropriate modules can be developed depending on available hardware. In particular, this study uses a SimCraft three-axis motion-based driving simulator.

## 2. Materials and Methods

**Figure 1:**
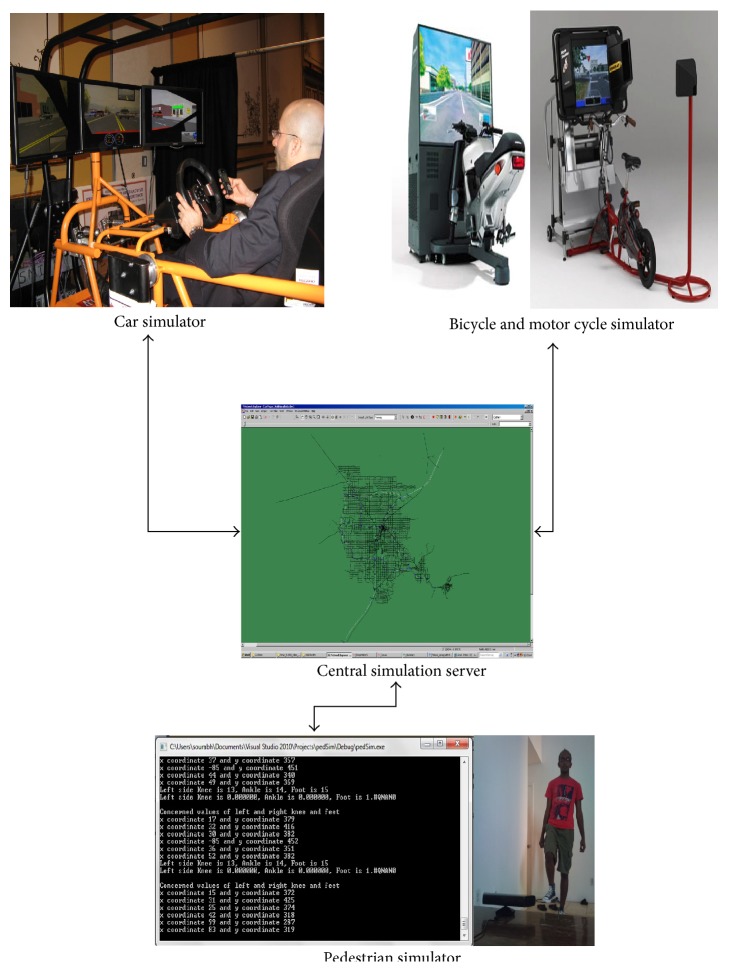
System architecture of n-MIPEDS. (The images for the bicycle and motorcycle simulators are used here for illustrative purposes only. Copyright: Honda.)

**Figure 2:**
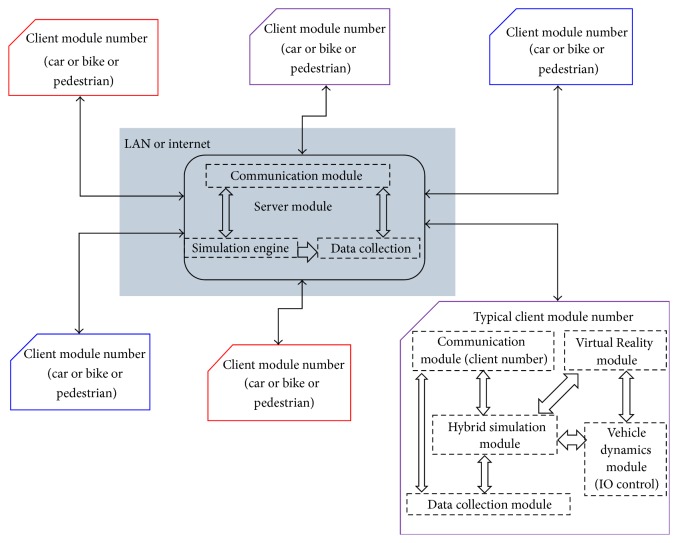
Data flow diagram.

The proposed architecture uses a multiplayer framework where each player is connected to the traffic system through a communication network (LAN or internet). The corresponding system architecture diagram and data flow diagram are shown in Figures 1 and 2, respectively.  Figure 1 illustrates the following:

1. *A Car Simulator*. This simulator generates motion and the corresponding forces based on the roadway conditions and the driver's behavior and actions. Hence, the human being driving in the driving simulator experiences a realistic journey and reveals a behavior that is not artificially affected by unrealistic modeling. This driver is termed in this study the virtual driver. In the experimental framework, a SimCraft motion-based driving simulator with 3 Degrees of Freedom (3DoFs) is used.
2. *A Pedestrian Simulator*. This simulator comprises Microsoft Kinect to capture and display the movements of walking human.
3. *A Bicycle and Motorcycle Simulator*. This is a motion-based simulator with capabilities to simulate a self-powered or fuel-powered bike. It consists of a computer CPU with a graphics card, a Head-Up-Display setup or LCD screen, a motion base, and a joystick.
4. *A Central Simulation Server*. This server runs simultaneously on parallel CPUs the mesoscopic and microscopic simulations and provides the background traffic to the driving, pedestrian, and bike simulators. It also takes care of the communication and data transfer between the different simulators. The implementation involves a high end supercomputer running Microsoft Windows 7 64-bit edition and networking hardware including a 1000BaseT Gigabit Ethernet as well as the necessary routers and switches to complete the network.

The data flow diagram illustrated in Figure 2 includes a server module and client modules. There are various types of clients to represent different traffic entities such as cars, bikes, or pedestrians. Each client module in turn includes the following modules:

- *Hybrid Simulation Module*. This hybrid simulation module is required to provide realistic and consistent traffic around the vicinity of the virtual driver and to capture the consequences of the driver actions on the entire system. It combines a microscopic and a mesoscopic traffic flow simulators. In this module, the actions and location of the virtual driver are directly and continuously incorporated into the microscopic model. The microscopic simulator continuously receives traffic from and sends traffic to the mesoscopic simulator considering the boundary established by the location of the virtual driver.
- *Virtual Reality (VR) Module*. This module creates a simulated world around the vicinity of the virtual driver and provides the associated audio and 3D graphics. It receives information from the hybrid simulation module and provides information to the virtual driver.
- *Vehicle Dynamics Module*. This module generates the motion related information for each of the simulators based on the traffic conditions and the users' actions.
- *Communication Module*. This module enables communication between the server and the client module. It is responsible for synchronizing the transfer of information between the client and the server. For various reasons, the modules proposed in this architecture are being developed using different environments. Hence, the Common Object Request Broker Architecture (CORBA) is to enable interoperability across different language and platforms [24].
- *Data Collection Module*. This module collects a vast array of data including drivers' and pedestrians' behavior as well as the associated traffic characteristics.

The server module integrates all the information and it is responsible for tracking the entire system performance using a communication and data collection module and a simulation engine. The simulation engine receives information from all the clients via the communication. That information is stored via the data collection module. Information about system states is also sent to each of the clients using the communication module.

### 2.1. Roadway Network Geometry

Some simulators such as STISIM Drive [13] only support modeling of corridors without enabling network-level representation. The proposed architecture enables the modeling of generalized networks. Geometric and control characteristics are particularly important for microscopic traffic flow simulation. Data about actual road network geometry for a given city can be obtained from Open Street Maps (OSM) [19] in  .xml format. This type of data includes latitude, longitude, street names, intersection details, and horizontal curve information. The OSM data is an open source of world maps maintained by users across the globe. It includes all the freeways, major roads, and many minor streets of every major city. The OSM data can be used to generate the network of roads for the Virtual Reality module in Figure 2. Missing data, such as lane information, traffic control, and signal timings, needs to be obtained from local or state agencies or any existing model.

**Figure 3:**
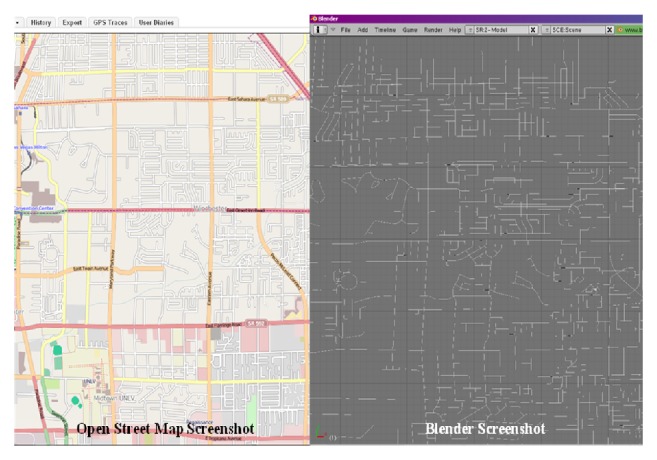
Generated network on Blender using Open Street Map.

The proposed architecture can be used for any network with the required information. In this study and for demonstration purposes, the Las Vegas road network was created using OSM data. Lane data, traffic control, and signal settings were obtained from an existing traffic simulation model. To obtain the correct mapping, the coordinates from OSM were matched with those in the existing model. A portion of the roadway network created using this approach is shown in Figure 3. This approach reduces the time required to generate a roadway network for the proposed architecture. Details about this approach are discussed below in Section 2.2.

### 2.2. Hybrid Simulation Model

Most driving simulators provide background traffic around the virtual driver using survey data and hourly volumes/distributions [13] or the desired traffic density [25, 26]. This approach has limitations to capture congestion related phenomena such as spillback and spillover. Microscopic simulation can be used to capture congestion related phenomena. However, the computational burden and the simulation time can increase significantly with network size. In addition, data needs and modeling time are prohibitively expensive for large-scale micro simulation models.

In order to adequately represent both the microlevel vehicle dynamics around the virtual driver and the traffic dynamics in the rest of the network, a hybrid simulation model that integrates a microscopic and a mesoscopic traffic flow simulation model is proposed. Félez et al. [27] have envisaged the required environment by integrating a driving simulation engine, SCANeR II, and a microscopic traffic-flow simulation model, AIMSUN. The authors have discussed and proposed solutions for various integration issues such as the matching of roadway geometry, computing speed for data exchange, simulation step size, and autonomous vehicle visualization. The paper also discusses some limitations of the integration including issues with control of the autonomous vehicles, vehicle kinematics in curve sections, and lane changing behavior. The proposed architecture in this study envisions higher consistency at a lower computation cost by integrating various simulators as interdependent components in a single framework. To the best of our knowledge, only Olstam et al. [28] have used a hybrid simulation model to provide background traffic to a driving simulator. The area surrounding the virtual driver was divided into one inner region and two outer regions. Vehicles in the inner region were simulated according to a microscopic model, while vehicles in the outer regions were updated according to a mesoscopic model. The authors mentioned that further research is required to address the following issues: (i) arterials and freeways with three or more lanes; (ii) ramps on freeways and intersections on rural roads; (iii) simulation of urban traffic conditions; and (iv) simulation on roadway networks.

Critical aspects in the development of hybrid simulation models are the compatibility of two different traffic flow streams and the propagation of traffic conditions at the interfaces [29]. Traffic propagation at interfaces should be analyzed both at free-flow conditions and at congested conditions. In mesoscopic models, vehicles move in an aggregate fashion while, in microscopic models, vehicles move according to individual vehicle dynamics. Hence, at the interfaces of meso to micro and micro to meso, traffic propagation both upstream and downstream has to be considered. It is necessary to define the time when both the models will transfer data based on the updating time intervals for the two models [30].

The proposed hybrid simulation model integrates the DynusT© [21], a mesoscopic simulation-based dynamic traffic assignment model, with a microscopic simulation model. The microscopic model includes a car-following model, a lane changing model, and a gap-acceptance model. Background traffic around the virtual driver is provided by the micro simulation model. Hence, the region covered by the micro simulation, called the *μ*Sim zone, is defined by the position of the virtual driver. The following methods can be used to define this region:

1. *Method 1 (Moving μSimlink).* Use the microscopic simulation model only on the roadway link where the virtual driver is present. Show the Virtual Reality Environment to the extent of the driver's visibility limit. Use mesoscopic simulation on all the other links. Thus, the *μ*Sim zone is the link where the virtual driver is located and it changes according to the position of the virtual driver.
2. *Method 2 (Moving μSimzone).* Use the microscopic simulation model in a zone around the position of the virtual driver. Show the Virtual Reality Environment to the extent of the driver's visibility limit. Use mesoscopic simulation on links other than those modeled using micro simulation. Thus, the *μ*Sim zone is the fixed zone with respect to position of the virtual driver.

In both methods, the problem of conserving vehicles should be solved at the boundary of mesoscopic and microscopic integration. In each simulation interval of the mesoscopic model, the entire network is updated based on the mesoscopic logic and the states at the boundary between the meso and micro models. However, the network states covered by the micro simulation model govern that zone.

### 2.3. User-Driven Vehicle Dynamics Model

The objective here is to generate actual vehicle motion dynamics so as to enhance modeling realism. This motion produces physiological and psychological reactions similar to those present in a real-world driving experience. Motion-axis simulators can be used to generate vehicular motion dynamics using DOFs ranging from two to fourteen [8–14, 25]. Implementation should be based on the required aspects for the particular problem context in order to avoid unnecessary computations.

Vehicle motion dynamics are generated using SimCraft 3DOFs motion-based simulator. Although 6DOFs are desired to reproduce most vehicle dynamics, the 3DOFs can realistically reproduce the most important motions such as the effects of acceleration/deceleration as well as changes on roadway geometry. The motion dynamics are generated based on the actions of the virtual driver, the geometric characteristics of the roadway, and the interactions with other vehicles. Hence, motion dynamics must be seamlessly synchronized with the Virtual Reality module.

### 2.4. Pedestrian, Bicycle, and Motorcycle Simulator

Existing pedestrian models [31, 32] require detailed data to capture the interactions between pedestrians and vehicles. Some studies [33] have focused on the study of pedestrian and driver behavior at crosswalk locations, where pedestrians and vehicles often interact. Numerous data has been collected about both pedestrians and drivers. However, the data collection process can be expensive and limited by the physical and operational characteristics of the location where the data is being collected. Hence, it is difficult to analyze multiple alternatives and the effects of critical factors. Pedestrian simulators enable circumventing some of these issues. The University College London has a Pedestrian Accessibility and Movement Environment Laboratory (PAMELA) [34] to study pedestrian movements under various environmental conditions. PAMELA has been used to better understand roadway aspects that affect pedestrian's ability to navigate the traffic system. The proposed pedestrian, bicycle, and motorcycle simulators have the unique capability of interacting together with a car or driving simulator. This capability enables studying a broad range of transportation phenomena using actual human beings in a safe and controlled environment.

Sensors can be used to capture the movements of various entities as well as human behavior including, for example, a pedestrian observing traffic while crossing a street and the walking speed relative to the traffic conditions. Traffic safety is highly influenced by users' behavior and their interactions. According to a report by the National Highway Traffic Safety Administration (NHTSA) [35], every year at least 50% of the motorcycle fatal crashes involve multiple vehicles; of that percentage, 41% had a blood alcohol concentration of 0.08 g/dL or higher. Safety is a primary concern not only for cars, but also for motorcycles and pedestrians. The proposed simulation framework enables the study of traffic safety for all these users.

The pedestrian simulator consists of a state-of-the-art Natural Interaction Sensor (Microsoft Kinect) and a head-mounted display. The movements of human walking are captured using the Microsoft Kinect. The Kinect consists of sensors that identify joints, body structure, facial features, and voice. The head-mounted display is to project the traffic conditions for the pedestrian. These traffic conditions are obtained using the communication module in the pedestrian simulation client.

### 2.5. Virtual Reality Environment

The Virtual Reality Environment is used to provide all the background as well as the traffic conditions representing the real-world to the various human beings navigating the system using the proposed simulators. The Virtual Reality Environment includes seven different components: simulation, interaction, artificiality, immersion, telepresence, full-body immersion, and network communication [36]. These components are used to provide an immersive traffic experience subject to hardware and software limitations.

In this study, the Virtual Reality Environment is created using 3D models [14]. To accelerate the modeling process and to achieve cost-effective development, an automated modeling process is required. A challenging problem for automation is creating and deploying 3D models at the required exact locations without deforming their sizes and shapes. Here, a hierarchical multilayer and data-driven approach is proposed. Each layer includes different types of objects which are recreated using data obtained from various sources.

**Figure 4:**
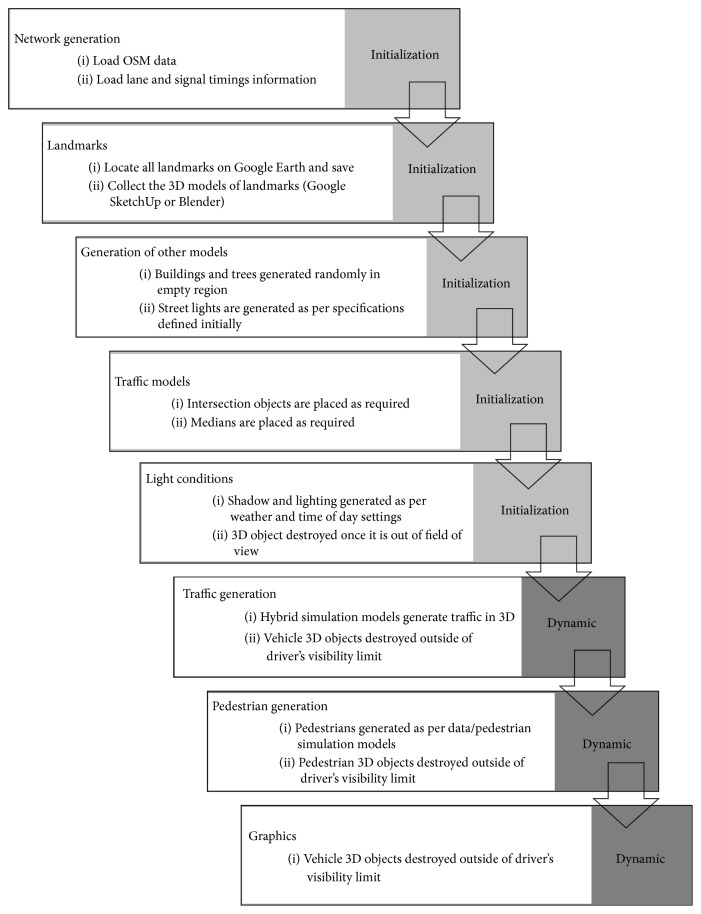
Layer-architecture for Virtual Reality generation.

Figure 4 illustrates the proposed multilayer approach for the generation of the Virtual Reality Environment. A list of landmarks is created and imported from Google Earth. Similarly, 3D images for the imported list are obtained from Google SketchUp [37] or created in Blender [20]. Landmarks are used to provide a perception of familiarity in the Virtual Reality Environment. The location of these models is automated using their latitudes and longitudes. The locations of other objects including trees and buildings and such roadside components as mailboxes, water pumps, fire hydrants, bus stop shelters, and street lights are defined as realistic as possible.

The Virtual Reality Environment is generated only to include the visibility limits for the virtual driver(s) and virtual pedestrian(s). The generated Virtual Reality Environment includes pedestrians as well as different classes of vehicles, such as cars and trucks. Different levels of visibility are available according to weather and time of day conditions. These conditions are recreated using various rendering techniques such as shading and reflecting.

### 2.6. Results and Discussion

One of the primary objectives of the proposed architecture is the collection of data about the vehicles, the users, and the system performance. Vehicle data includes variables such as lateral position, vehicle trajectories, vehicle heading angle, acceleration/deceleration, and braking times. Users' data includes variables such as perception-reaction times, physiological data obtained from electrocardiograms, galvanic skin response, and body temperature. The hybrid simulation model will collect system performance data. The data collection module is included in every single client so as to collect the corresponding information.

The initial implementation of the proposed architecture began with control of the driving simulator using the Software Development Kit (SDK) provided by the manufacturer, SimCraft. A control module was created using a Dynamic Link Library (.dll) file provided in SDK. Blender [20] was chosen for the development of graphics because it provides the required minimum capabilities. Blender is open source and has a big user base and a support community. In addition, Blender [20] is supported in both Linux and Windows. Once the graphics of roadway network were developed, a vehicle model was created to drive in the network. The scripts developed in this study can be used to generate models of the transportation network of any city. This requires using Open Street Maps.

Microsoft Kinect is currently being used for the development of the pedestrian simulator. A walk identification module has been created with a text output. This will later be developed and integrated into the pedestrian simulator. Blender [20] is used for creating 3D models for various transportation components, such as different roadways, traffic signal displays, buildings, and trees. The proposed architecture requires views inside the Virtual Reality Environment for cars, motorcycles, bicycles, and pedestrians. Multiple CPUs are used to generate these views. A networking module was developed to enable data transfer across the multiple CPUs. CORBA is used for this purpose along with omniORB C++ and Python.

Future work includes (i) the implementation of hybrid simulation model for the driving and pedestrian simulators; (ii) the graphics module for Virtual Reality Environment; (iii) the development of hardware for bicycle and motorcycle simulator; and (iv) the integration, coordination, and synchronization of all the components of the proposed architecture.

## 3. Conclusions

This study has proposed an architecture for an interactive and motion-based simulation of a vehicular and pedestrian traffic system. The proposed architecture increases the realism of existing alternative modeling approaches by explicitly and simultaneously including actual drivers, pedestrians, and bikers. In addition, the architecture enables the modeling of the entire network with reasonable investment of resources. To the best of our knowledge, there is no alternative architecture that simultaneously considers all the elements of reality considered here. Existing modeling frameworks focus on a particular component of the real-world system; the reaming components are ignored or modeled using artificial entities. State-of-the-art modeling and analysis tools such as simulation-based Dynamic Traffic Assignment, CORBA, and Open Street Maps enable the implementation of the proposed architecture. Implementation of the architecture will provide the unique capability to study countless traffic problems using actual human beings.
